# Spatiotemporal Assessment of Benzene Exposure Characteristics in a Petrochemical Industrial Area Using Mobile-Extraction Differential Optical Absorption Spectroscopy (Me-DOAS)

**DOI:** 10.3390/toxics13080655

**Published:** 2025-07-31

**Authors:** Dong keun Lee, Jung-min Park, Jong-hee Jang, Joon-sig Jung, Min-kyeong Kim, Jaeseok Heo, Duckshin Park

**Affiliations:** 1 Nakdong River Basin Environment Office, Changwon-si 51439, Republic of Korea; ldk0225@korea.kr (D.k.L.); anspark011@korea.kr (J.-m.P.); jhjang1013@korea.kr (J.-h.J.); 2 R&D Strategy Department, Korea Railroad Research Institute (KRRI), Cheoldo Bangmulgwanro, Uiwang-si 16105, Republic of Korea; mkkim15@krri.re.kr; 3 Transportation Environmental Research Department, Korea Railroad Research Institute (KRRI), Cheoldo Bangmulgwanro, Uiwang-si 16105, Republic of Korea; jsheo1005@krri.re.kr

**Keywords:** VOCS, Me-DOAS, petrochemical complex, three-dimensional monitoring

## Abstract

Petrochemical complexes are spatially expansive and host diverse emission sources, making accurate monitoring of volatile organic compounds (VOCs) challenging using conventional two-dimensional methods. This study introduces Mobile-extraction Differential Optical Absorption Spectroscopy (Me-DOAS), a real-time, three-dimensional remote sensing technique for assessing benzene emissions in the Ulsan petrochemical complex, South Korea. A vehicle-mounted Me-DOAS system conducted monthly measurements throughout 2024, capturing data during four daily intervals to evaluate diurnal variation. Routes included perimeter loops and grid-based transects within core industrial zones. The highest benzene concentrations were observed in February (mean: 64.28 ± 194.69 µg/m^3^; geometric mean: 5.13 µg/m^3^), with exceedances of the national annual standard (5 µg/m^3^) in several months. Notably, nighttime and early morning sessions showed elevated levels, suggesting contributions from nocturnal operations and meteorological conditions such as atmospheric inversion. A total of 179 exceedances (≥30 µg/m^3^) were identified, predominantly in zones with benzene-handling activities. Correlation analysis revealed a significant relationship between high concentrations and specific emission sources. These results demonstrate the utility of Me-DOAS in capturing spatiotemporal emission dynamics and support its application in exposure risk assessment and industrial emission control. The findings provide a robust framework for targeted management strategies and call for integration with source apportionment and dispersion modeling tools.

## 1. Introduction

Chronic exposure to chemical substances poses significant health risks to humans, particularly due to their potential for bioaccumulation and toxicity. Among these, volatile organic compounds (VOCs) released into the atmosphere have shown a steady increase in emissions [1]. Major anthropogenic sources of VOCs include crude oil storage tanks, petrochemical facilities, and gasoline stations. In South Korea, in recognition of the adverse health and ecological effects associated with prolonged exposure to hazardous air pollutants (HAPs), the Ministry of Environment (MOE) has designated 35 toxic substances—such as benzene, styrene, and ethylbenzene—as specific air pollutants subject to regulatory control [2].

Benzene, classified by the International Agency for Research on Cancer (IARC) as a Group 1 carcinogen, is a well-documented air pollutant with established health risks through inhalation exposure [3]. It is primarily emitted during crude oil refining and chemical manufacturing processes involving plastics and synthetic fibers and may also be released during industrial accidents, thereby posing significant exposure risks to nearby communities [4].

The major health outcomes associated with benzene exposure include acute myeloid leukemia (AML), acute lymphocytic leukemia (ALL), non-Hodgkin lymphoma (NHL), chronic lymphocytic leukemia (CLL), multiple myeloma (MM), chronic myeloid leukemia (CML), childhood AML, and lung cancer [5,6,7,8,9,10,11,12,13].

Since 2010, South Korea has enforced an annual ambient air quality standard for benzene of 5 µg/m^3^. However, most previous studies on HAPs and VOCs, including benzene, have relied on point-based measurements conducted at fixed monitoring sites within industrial complexes. These conventional approaches are limited in their ability to capture real-time temporal and spatial variations. Due to their high volatility and reactivity, VOCs are easily dispersed under varying environmental conditions, highlighting the need for real-time exposure monitoring to support effective risk assessment.

Traditional VOC sampling techniques—such as the use of adsorbent tubes, Tedlar bags, and cartridges—require subsequent laboratory analysis using gas chromatography–mass spectrometry (GC/MS) or high-performance liquid chromatography (HPLC). These methods introduce significant time delays and offer limited temporal resolution.

To address these limitations, real-time mobile monitoring systems such as Selective Ion Flow Tube Mass Spectrometry (SIFT-MS) have recently been applied in atmospheric VOC analysis. However, SIFT-MS is challenged by its limited ability to distinguish between isomeric compounds or substances with similar molecular weights and is prone to high background signals, necessitating rigorous quality assurance protocols [14].

Mobile-extraction Differential Optical Absorption Spectroscopy (Me-DOAS), an optical remote sensing technique based on light absorption, offers a promising alternative. Unlike conventional methods such as GC/FID, Me-DOAS allows for real-time detection of VOCs without the need for sample preparation. When integrated with meteorological sensors for wind speed and direction, Me-DOAS facilitates on-the-move identification of benzene plumes and their dispersion dynamics [15].

South Korea is home to approximately 1300 industrial complexes, including three major petrochemical hubs: Ulsan, Yeocheon, and Daesan. Due to unplanned urban development, many of these complexes are located near residential areas, thereby increasing the potential for human exposure. Given their large spatial extent and heterogeneous emission sources, conventional two-dimensional monitoring techniques face significant limitations in identifying and characterizing emission hotspots.

In this context, the present study aims to assess benzene emissions using a comprehensive spatiotemporal framework by employing Me-DOAS integrated with a Solar Occultation Flux (SOF) system within the Ulsan Petrochemical Complex—one of South Korea’s largest industrial zones. To the best of our knowledge, this is the first study to apply Me-DOAS for a time-series assessment of benzene exposure in this industrial area. The findings provide a scientific basis for understanding the temporal and spatial distribution of benzene and for developing more effective, targeted management strategies.

## 2. Materials and Methods

### 2.1. Study Area and Measurement Approach

**Figure 1:**
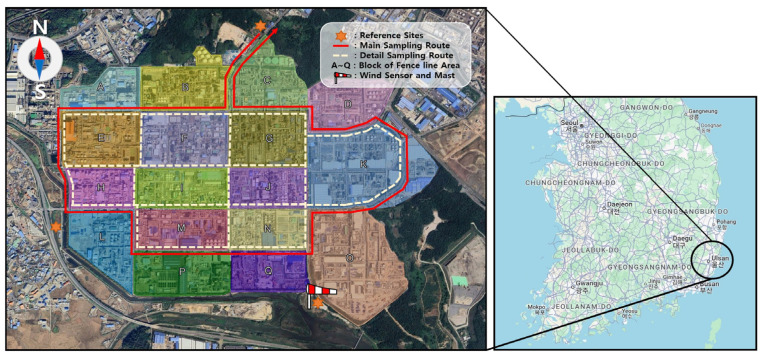
Geographic overview of the study area: Ulsan Petrochemical Industrial Complex. The complex spans approximately 4.8 km^2^ and includes a mix of petrochemical, automotive, and shipbuilding facilities. Residential and commercial zones are located approximately 1 km southwest of the industrial boundary.

The study area encompasses the Ulsan Petrochemical Industrial Complex, which spans approximately 4.8 km^2^ and contains around 700 industrial facilities, including petrochemical, automotive, and shipbuilding companies (Figure 1). Within a 1 km radius southwest of the complex lies a mixed residential and commercial zone, while the surrounding areas are predominantly other industrial districts. Ulsan is known for frequent public complaints regarding odorous emissions—particularly during nighttime hours—and reports relatively low public satisfaction with air quality (37.7%) [16]. As one of South Korea’s largest petrochemical hubs, Ulsan ranked fourth nationwide in VOC emissions in 2020, with approximately 820,000 tons/year released [17]. Measurements were conducted monthly from January to December 2024. On each sampling day, four measurement sessions were conducted to capture diurnal variations: morning (10:00–12:00), afternoon (13:00–15:00), nighttime (22:00–24:00), and predawn hours (01:00–03:00 LT).

**Figure 2:**
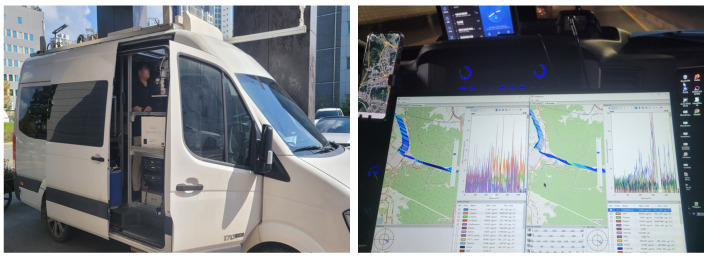
Mobile measurement campaign using the Me-DOAS system. Monitoring was conducted along outer boundary roads in a counterclockwise direction and repeated multiple times per session. High-resolution grid-based measurements were also performed within the core area of the industrial complex.

The mobile monitoring route followed the outer roadways of the complex in a counterclockwise direction and was repeated at least three times per session to ensure data consistency and reliability. In addition, high-resolution, grid-based measurements were performed within the interior of the complex to capture fine-scale spatial variations (Figure 2).

Meteorological conditions were monitored at the measurement sites using a wind sensor and mast system (WindSensor and Mast, FluxSense Co., Ltd., Stockholm, Sweden) installed in an unobstructed open area to minimize interference from nearby structures. For comparative analysis, three fixed reference sites were established. The upwind reference site was dynamically selected for each session based on the prevailing wind direction.

This study adapted the U.S. Environmental Protection Agency’s (EPA) fence line monitoring approach for benzene to the specific characteristics of the study area. The fence line technique integrates concentration measurements with meteorological parameters (i.e., wind speed and direction) at the boundary of the site to estimate the potential origin of emissions [18].

### 2.2. Measurement and Analytical Methods

Measurements in this study were conducted using a Mobile-extraction Differential Optical Absorption Spectroscopy (Me-DOAS) system developed by FluxSense Co., Ltd., Stockholm, Sweden, mounted on a vehicle. Me-DOAS is an ultraviolet (UV)-based optical instrument capable of detecting volatile organic compounds (VOCs), including BTEX compounds—benzene, toluene, ethylbenzene, and xylene. The system employs an internal UV light source and a White Cell mirror configuration with a total optical path length of 210 m, enabling high-sensitivity detection. Spectral analysis was performed in the UV wavelength range of 250–275 nm using a Me-DOAS system equipped with a Czerny–Turner grating spectrometer, which provides a spectral resolution of approximately 0.15 nm. This high resolution enables reliable discrimination of BTEX compounds through their characteristic UV absorption features. For benzene, the instrument achieves a detection limit of approximately 1 ppb, with both measurement accuracy and precision around ±10% under typical operating conditions [15].

**Figure 3:**
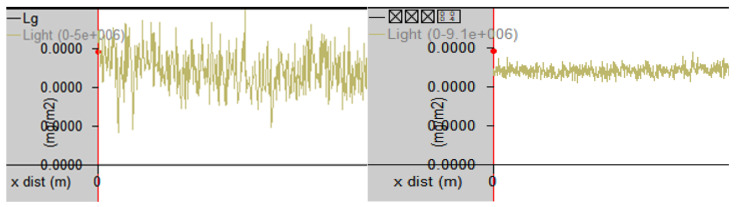
Instrument stabilization procedure for the Me-DOAS system. (Left: unstable light source condition; Right: stabilized light source condition prior to measurement; Lg stands for Light Path Length).

To ensure reliable measurements, the vehicle maintained a driving speed below 20 km/h. GPS location and timestamps were continuously recorded via FluxSense’s mapping system. Prior to each measurement session, the instrument underwent a stabilization phase at an upwind baseline site for at least 40 min to ensure consistent light source output and overall system stability (Figure 3).

The validity of the Me-DOAS measurements has been supported by previous intercomparisons with gas chromatography–flame ionization detection (GC-FID) analyses of canister samples, which demonstrated less than 10% deviation in BTEX concentrations [15]. Furthermore, during the 2013 DISCOVER-AQ campaign in Houston, Me-DOAS data showed strong agreement with Proton Transfer Reaction Mass Spectrometry (PTR-MS) results [19]. In the present study, additional validation was performed through parallel operation with a Selective Ion Flow Tube Mass Spectrometry (SIFT-MS) system over a one-year period. The two systems exhibited consistent peak patterns at corresponding locations, further corroborating previous validation findings [20].

To refine the dataset, raw values were cross-checked against real-time mapping outputs, and non-representative data segments—such as those recorded during vehicle stops or instrument anomalies—were excluded. The filtered dataset was processed into secondary analysis files (Excel format) to quantitatively assess benzene exposure levels across the study area.

High-concentration “hot spot” events were identified based on an exceedance threshold of 30 µg/m^3^. Real-time wind speed and direction data were used to confirm the potential alignment of emission sources. This threshold was selected with reference to the U.S. EPA Integrated Risk Information System (IRIS) chronic reference concentration (RfC) for occupational inhalation exposure—associated with decreased lymphocyte counts—and is also consistent with Korea’s indoor air quality guidelines [21,22].

### 2.3. Data Analysis

**Figure 4:**
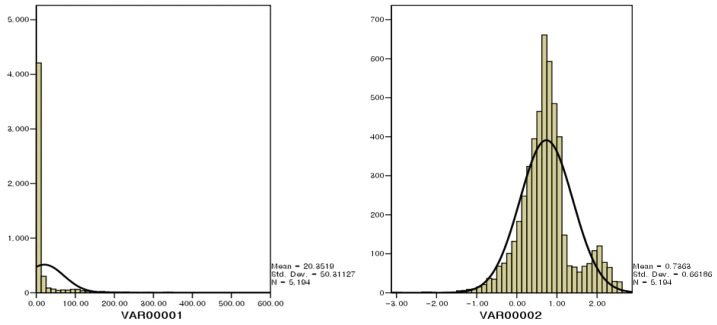
Log-normal distribution of benzene concentrations measured at the Ulsan Petrochemical Industrial Complex. The data exhibit a right-skewed distribution pattern, supporting the use of log-transformed statistical analyses.

Statistical analyses were performed using SPSS version 12.0 (SPSS Inc., Chicago, IL, USA) and SAS version 8.2 (SAS Institute Inc., Cary, NC, USA). A normality test of the measured benzene concentrations indicated that the data followed a log-normal distribution (Figure 4). To characterize the distribution of benzene concentrations, descriptive statistics were calculated, including arithmetic mean, standard deviation (SD), minimum and maximum values (range), geometric mean (GM), and selected percentiles.

Comparative analyses of benzene concentrations by month and measurement session were conducted using independent *t*-tests and one-way analysis of variance (ANOVA) to determine statistical significance. Pearson correlation analysis was employed to examine the relationship between the frequency and magnitude of high-concentration benzene “hot spots”, the number of stacks involved in benzene-handling processes, and total estimated emissions.

Spatial visualization of high-concentration benzene zones was carried out using a geographic information system (GIS) platform based on Google Earth (Google LLC, Mountain View, CA, USA).

### 2.4. Measurement Limitations and Data Quality Assurance for ORS Instruments

The optical remote sensing (ORS) technique quantifies atmospheric gas concentrations by analyzing open-path absorption spectra. While ORS provides rapid, non-intrusive, and spatially resolved measurements, its reliability can be challenged when target gas concentrations are near background levels or within trace ranges. Under such conditions, the signal-to-noise ratio (SNR) may fall below acceptable thresholds, introducing statistical uncertainty due to random fluctuations and white noise.

To ensure data integrity, we implemented a multi-tiered quality assurance protocol. First, negative values occurring under low-SNR conditions were classified as below the method detection limit (MDL) and excluded from analysis. Second, isolated concentration peaks lacking directional or temporal correlation with known emission sources were identified as false positives and cross-validated using concurrent meteorological data, including wind direction, speed, and PM levels. Third, time series showing erratic fluctuations without environmental causality were discarded unless repeatable trends were observed across multiple scans. Instrumental conditions—such as insufficient warm-up time, thermal drift, or optical misalignment—were also assessed during data screening.

To minimize interpretive bias, all data were first analyzed by a trained primary analyst and independently reviewed by a secondary expert. This dual-review process aligns with established QA/QC guidelines and ensures analytical robustness.

For field measurements, we employed a mobile DOAS (Me-DOAS) system equipped with an internal light source to detect aromatic VOCs, including benzene, across the Ulsan Petrochemical Complex. While Me-DOAS is highly effective for gases such as NO_2_ and SO_2_, its application to benzene is constrained by weak absorption features and spectral interferences. As a result, the benzene concentrations reported herein should be regarded as semi-quantitative, and this characteristic may lead to potential under- or overestimation of exposure levels. Despite this limitation, the collected data were robust enough to characterize spatiotemporal exposure patterns—capturing variations by location, time of day, and month—and to yield critical insights for identifying high-risk zones and supporting targeted exposure mitigation strategies.

### 2.5. Benzene Exposure Standards

In South Korea, benzene is classified as a toxic substance and is strictly regulated under the Act on the Registration and Evaluation, etc., of Chemical Substances (Article 42), requiring prior import notification and registration. According to the Framework Act on Environmental Policy, the annual ambient air quality standard for benzene is set at 5 µg/m^3^ to protect public health and ensure a pleasant living environment. In addition, the Indoor Air Quality Control Act stipulates a recommended guideline of 30 µg/m^3^ or less for newly constructed multi-use residential buildings to maintain healthy indoor air quality.

For occupational exposure, the Ministry of Employment and Labor has established exposure limits for benzene; the time-weighted average (TWA) is 0.5 ppm for an 8 h workday, while the short-term exposure limit (STEL) is 2.5 ppm, based on a 15 min exposure during a standard workday (8 h/day, 5 days/week). The Ministry of Food and Drug Safety also recommends limiting occupational exposure to below 2 ppm [23,24].

Internationally, benzene exposure standards in ambient air can be broadly categorized into three groups based on a threshold of 5 µg/m^3^ (Table 1). Benzene air quality standards vary considerably across countries and can be broadly categorized into three groups based on their annual allowable concentrations. Category 1 includes countries with stringent standards below 5 µg/m^3^, such as France (2 µg/m^3^), Israel (1.3 µg/m^3^), and Japan (3 µg/m^3^). Category 2 comprises countries that have adopted a 5 µg/m^3^ threshold, including India, Russia, South Korea, the European Union (EU), and Colombia. In contrast, Category 3 represents countries with more lenient standards exceeding 10 µg/m^3^, such as Syria, Vietnam, and South Africa. Most countries adopt an annual average standard; however, some also apply 24 h or even hourly limits. For instance, Russia enforces an 8 h limit of 100 µg/m^3^ and a 24 h limit of 300 µg/m^3^. Vietnam has a 1 h standard of 22 µg/m^3^, while Cuba allows up to 1000 µg/m^3^ over a 10 min period [25,26,27,28,29,30].

## 3. Results and Discussion

### 3.1. Monthly Variation in Benzene Concentrations

Table 2 summarizes the monthly measurements of benzene concentrations in the Ulsan Petrochemical Industrial Complex. The highest arithmetic mean concentrations were observed in February, followed by January, April, and November (secondary campaign). Specifically, the arithmetic mean ± standard deviation in February was 64.28 ± 194.69 µg/m^3^ (GM: 5.13 µg/m^3^), in January 23.55 ± 80.33 µg/m^3^ (GM: 5.13 µg/m^3^), and in April 20.35 ± 50.31 µg/m^3^ (GM: 5.44 µg/m^3^). The arithmetic mean concentration in February was approximately 2.7 to 11.6 times higher than in other months, while the geometric mean in January was 1.4 to 2.7 times higher than in other months. Statistically, January exhibited significantly higher concentrations compared to other months (*p* < 0.01).

Although a direct comparison with the national ambient air quality standard (annual average of 5 µg/m^3^) is limited, it is notable that all 12 months exceeded this threshold based on arithmetic means, and 4 months (January, February, April, and November) exceeded it based on geometric means.

A seasonal analysis of benzene concentrations at the Yeosu industrial complex (2013–2014) reported the highest values during winter (2.73 ppb), followed by autumn (1.52 ppb), spring (1.39 ppb), and summer (0.49 ppb) [31]. Similar seasonal patterns—higher levels in winter and autumn and lower in summer—have been observed in other Korean studies [32,33,34]. Benzene levels in steel industry complexes were also found to peak during autumn [35]. The elevated concentrations in winter are likely attributable to lower atmospheric mixing heights, reduced dispersion, and increased heating-related emissions, whereas in summer, plant operations may decrease due to vacation periods, and higher temperatures may enhance photochemical transformation of VOCs [34,36].

**Figure 5:**
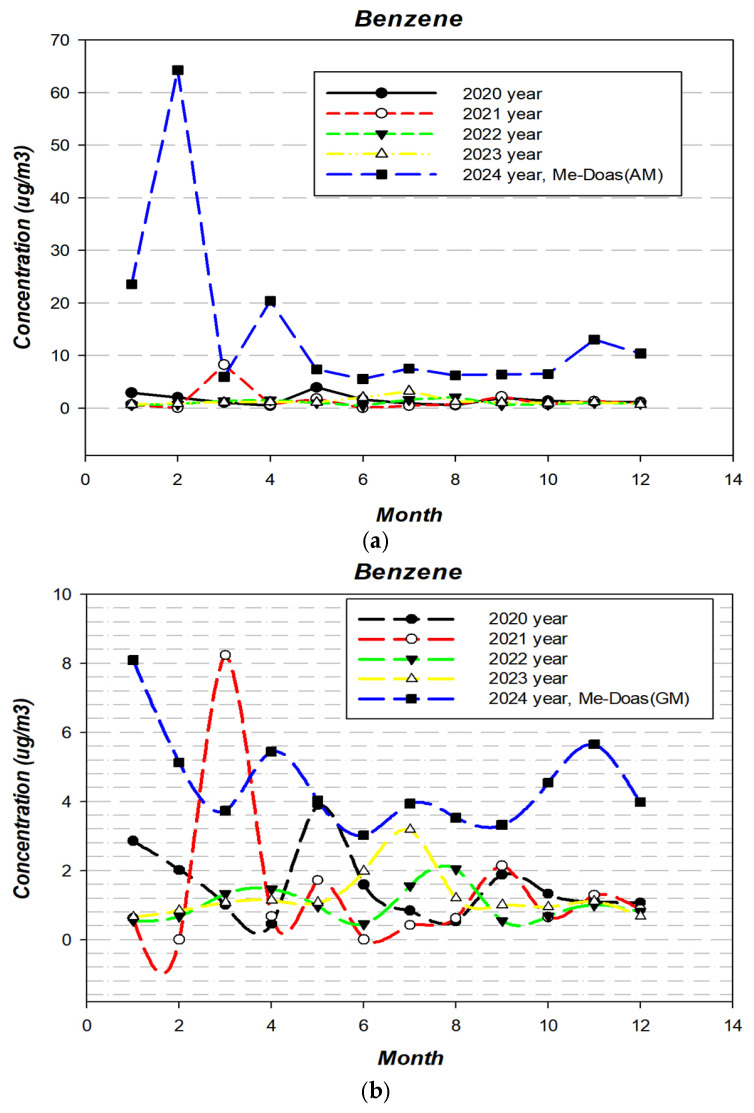
Comparison of benzene concentrations between the study area and the regulatory monitoring station. (**a**) Comparison with hazardous air pollutant monitoring station (based on arithmetic mean). (**b**) Comparison with hazardous air pollutant monitoring station (based on geometric mean).

To further assess monthly variation, benzene data from a hazardous air pollutant monitoring station located approximately 1.4 km northeast of the study site were analyzed for the years 2020–2023 (Figure 5). The maximum and minimum monthly averages at the monitoring site were as follows.

An analysis of the monthly average concentrations recorded at the monitoring site revealed notable inter-annual variations in peak and minimum levels. In 2020, concentrations ranged from a maximum of 3.89 µg/m^3^ in May to a minimum of 0.45 µg/m^3^ in April. In 2021, levels spanned from 8.23 µg/m^3^ in March to 0.61 µg/m^3^ in January. The year 2022 showed a narrower range, with a high of 1.56 µg/m^3^ in July and a low of 0.55 µg/m^3^ in both January and September. In 2023, the maximum concentration was 3.17 µg/m^3^ in July, while the minimum was 0.64 µg/m^3^ in January. Whereas our study showed elevated benzene concentrations during colder months, the nearby regulatory monitoring station reported relatively higher concentrations in warmer periods. This discrepancy is likely due to spatial separation (~1.4 km) between the monitoring locations and the different influence of environmental variables such as temperature, wind direction, and wind speed.

A study conducted in Busan’s industrial areas reported that on-site benzene concentrations were approximately twice as high as those recorded by the nearby regulatory station [37]. Similarly, higher total VOCs (TVOCs) were reported during winter and autumn compared to spring and summer, with photochemical reactions and precipitation likely contributing to reduced benzene levels during the summer [38].

In this study, both arithmetic and geometric means were compared with data from the fixed monitoring station. The station measures ambient benzene using 2 h integrated sampling, from which daily, monthly, and annual averages are derived. However, due to the intermittent and spatially heterogeneous nature of benzene emissions in petrochemical complexes, arithmetic means may overestimate or underestimate exposure depending on the presence of high or low concentration “hot spots” at specific times and locations. Therefore, rather than relying solely on arithmetic averages as representative values, we propose the use of geometric means as a more suitable indicator that accounts for the skewed distribution and episodic nature of benzene emissions.

### 3.2. Temporal Variation of Benzene Concentrations by Measurement Session

**Figure 6:**
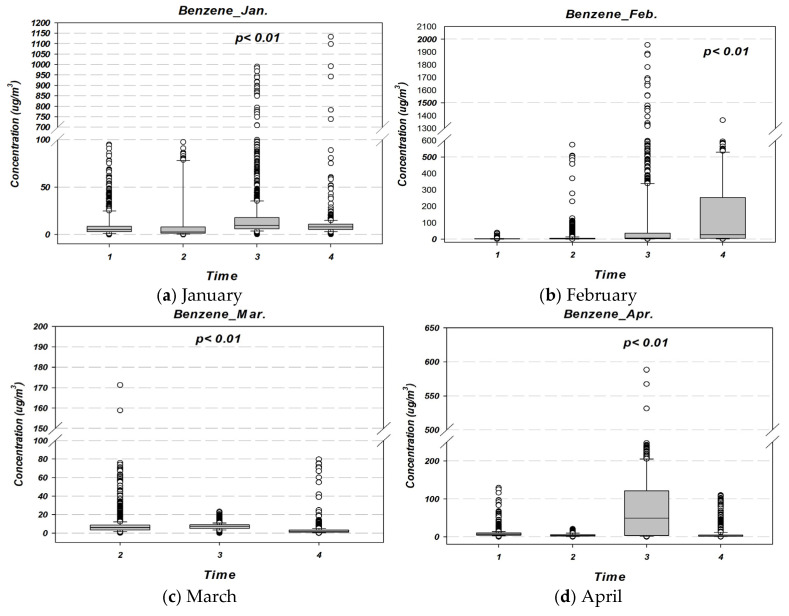

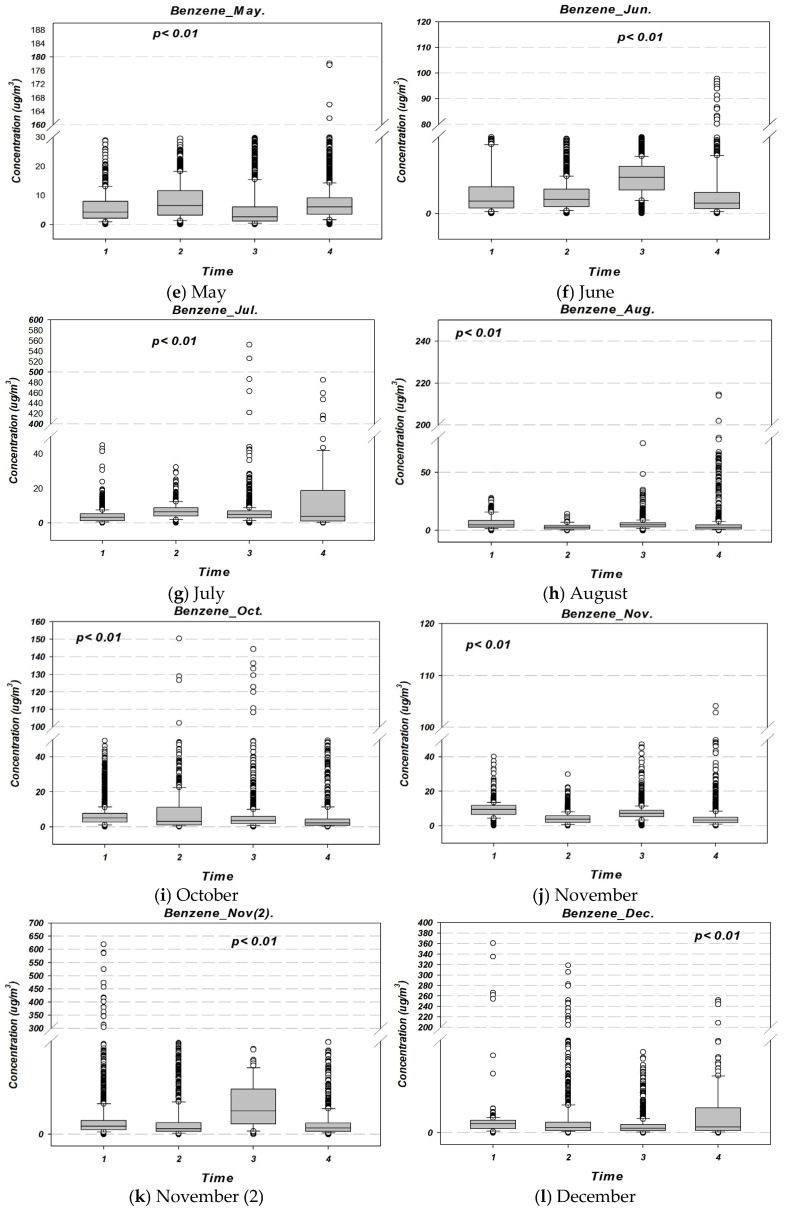
Benzene concentrations by time of day across different months in 2024 at the Ulsan Petrochemical Industrial Complex. The measurement periods were classified as follows: (1) morning (10:00–12:00); (2) afternoon (13:00–15:00); (3) nighttime (22:00–24:00); and (4) predawn hours (01:00–03:00 LT).

Figure 6 presents benzene concentrations measured at different times of day within the Ulsan Petrochemical Industrial Complex. Based on arithmetic mean values, the highest concentrations were observed during the fourth session in February, followed by the third session in February, the third session in April, and the fourth session in July. Specifically, the mean ± standard deviation for the 4th measurement session in February was 165.70 ± 248.41 µg/m^3^ (GM: 27.67 µg/m^3^), followed by 106.46 ± 260.43 µg/m^3^ (GM: 11.14 µg/m^3^) for the 3rd measurement session in February, and 80.20 ± 94.30 µg/m^3^ (GM: 21.85 µg/m^3^) for the 3rd measurement session in April.

In terms of geometric mean, the highest values were recorded as follows: the 4th measurement session in February (27.67 µg/m^3^) > the 3rd measurement session in April (21.85 µg/m^3^) > the 3rd measurement session in November (2nd campaign) (14.41 µg/m^3^) > the 3rd measurement session in February (11.14 µg/m^3^). The arithmetic mean during the 3rd measurement session in February was approximately 1.6 to 80.8 times higher than in other sessions, while the geometric mean was 1.3 to 17.2 times higher (*p* < 0.01), indicating statistically significant temporal variation.

Excluding May, July, August, October, and November (first campaign), most peak benzene levels were observed during the third and fourth sessions, which correspond to late-night and early-morning periods. This trend may be partially attributed to thermal inversions that limit vertical dispersion, as well as to operational factors specific to the chemical industry in Korea (e.g., nighttime transport or raw material handling). Further research is needed to clarify these associations.

A similar diurnal pattern has been reported by Shin et al. (2024), who found that benzene concentrations tend to increase during early morning hours, decline during the day, and rise again after sunset [39]. Prior research comparing daytime and nighttime VOC concentrations in industrial areas also found that nighttime levels were approximately 1.9 times higher than daytime levels [35]. Choi et al. (2009) likewise reported elevated nighttime benzene concentrations in Korea [40], and this pattern has also been observed internationally—in Yokohama, Japan (Tiwari et al., 2010); Barcelona, Spain (Filella & Peñuelas, 2006); and the eastern Himalayas, India (Sarkar & Khillare, 2014) [41,42,43].

This diurnal difference may result from more active atmospheric dispersion during the day, while VOCs emitted during the day may persist into the night due to reduced photodegradation. In addition, nighttime emissions continue to contribute to higher concentrations. Given that the atmospheric half-life of benzene is approximately 9.04 days [44], accumulation is likely in the absence of strong dispersive winds, especially during nighttime stagnation events.

### 3.3. High-Concentration Benzene “Hot Spot” Episodes

**Figure 7:**
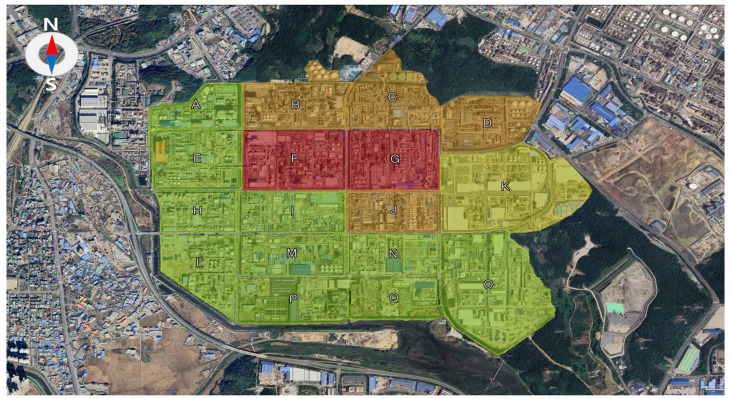
Detection frequency of high-concentration benzene “hot spots” in the Ulsan Petrochemical Industrial Complex. Blocks are color-coded by exceedance frequency of the 30 µg/m^3^ threshold: Red = >30 cases; Orange = 21–30 cases; Yellow = 11–20 cases; Green = ≤10 cases.

Figure 7 presents the number of exceedances of the benzene concentration threshold (30 µg/m^3^) at the Ulsan Petrochemical Industrial Complex. A total of 179 exceedance events were recorded during the measurement campaign. Among these, Block G exhibited the highest frequency, with 41 cases (23% of total), followed by Block F (32 cases), Block B (28 cases), Block J (25 cases), Block C (23 cases), Block D (25 cases), and Block K (14 cases).

In Block G, exceedances were most frequent during the fourth session (01:00–03:00), with 20 cases, followed by 11 cases in the third session, and five cases each during the first and second sessions. Block F showed 13 exceedances during the third session (22:00–24:00), 7 during the fourth, 4 in the second, and 9 in the first session. While direct comparisons must be interpreted cautiously, some locations, particularly Block B, exhibited benzene levels up to 400 times higher than the national annual standard (5 µg/m^3^), indicating the potential for localized high-exposure risk to on-site workers.

When analyzed by month, the highest number of exceedances occurred in February (33 cases), followed by November (second campaign, 30 cases), January (28 cases), and December (16 cases). These months, corresponding to the colder season in Korea, showed notably higher exceedance frequencies than other times of the year. In contrast, the total number of exceedances in summer months (June–August) was 23 cases, while spring (March–May) and autumn (September–October) recorded 30 and 19 cases, respectively.

For context, benzene concentrations measured in Ansan, Korea, were reported at 0.58 ± 0.33 ppb, while a downwind site near a petrochemical complex measured significantly higher levels at 3.1 ± 4.31 ppb [39]. Internationally, average concentrations in New Delhi, India, and Bangkok, Thailand, were 2.8 ppb and 5.8 µg/m^3^ (approximately 1.6 ppb), respectively. Comparatively, benzene levels observed in this study were several times higher in terms of geometric mean, and tens of times higher based on arithmetic mean.

Chae et al. (2024) reported average benzene concentrations across five Korean industrial complexes, including Ulsan (Onsan: 1.02 ppb), Daesan (0.78 ppb), Yeosu/Gwangyang (0.73 ppb), Po-hang (0.57 ppb), and Siwha/Banwol (0.51 ppb) [45]. Previous studies targeting industrial areas in Ulsan and Jinju similarly found significantly higher VOC concentrations compared to adjacent urban centers. These elevated levels were attributed to emissions from chemical handling processes and logistics operations involving the transport of raw materials [31,34,46].

**Figure 8:**
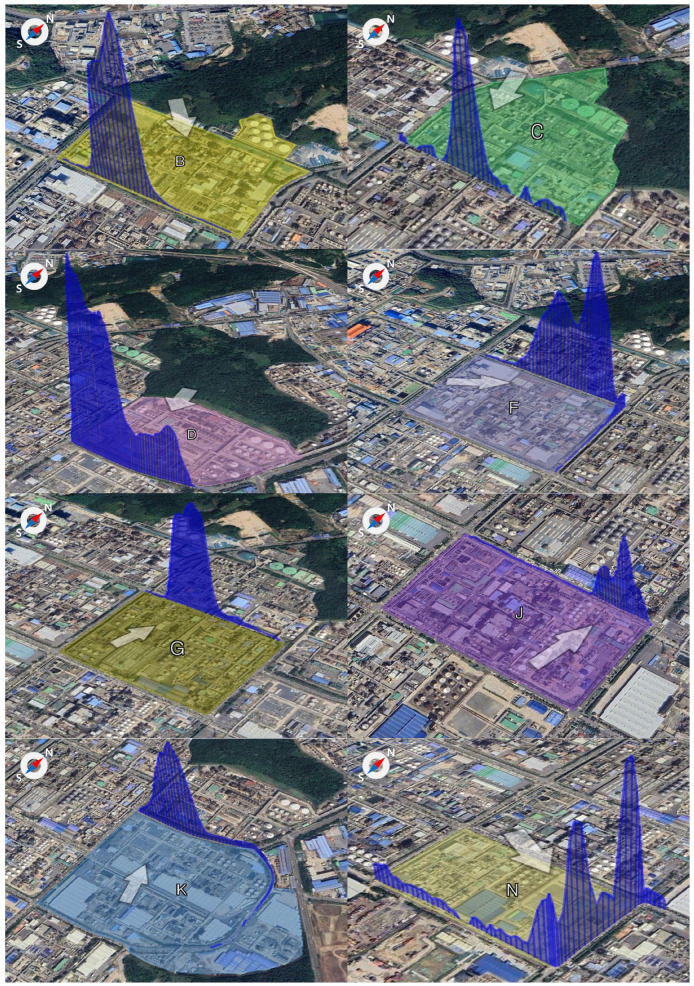
Benzene detection patterns by wind direction at each monitoring location within the Ulsan Petrochemical Industrial Complex (The arrows in the figure indicate wind direction).

The elevated number of exceedance events during winter months and during the third and fourth measurement sessions (evening and early morning) suggests a combined influence of industrial activity and meteorological factors. The primary cause of high-concentration episodes is likely the presence of benzene-handling facilities in the vicinity, while the secondary cause may involve accidental releases, leaks, and dispersion conditions such as wind direction and speed (Figure 8).

To effectively reduce benzene emissions and exposure risks, future control strategies must consider these environmental and operational factors. According to the U.S. EPA’s fence-line monitoring guidelines, facilities exceeding an annual benzene concentration of 9 µg/m^3^ are required to take mitigation measures [18]. Therefore, a tailored management framework that reflects the spatial, temporal, and seasonal characteristics of benzene emissions in the Ulsan petrochemical complex is warranted.

### 3.4. Analysis of Contributing Factors to High-Concentration Benzene Spot Occurrences

Table 3 presents a summary of potential influencing factors for benzene concentration in the Ulsan Petrochemical Industrial Complex, based on 2022 data from the Ministry of Environment’s Stack Emission Management System (SEMS). According to the analysis, benzene emissions tended to be higher in blocks with a larger number of processing facilities, particularly those involving storage and manufacturing operations.

The estimated benzene emissions by block revealed the following descending order: Block F (14,844.33 kg/year) > Block G (2819.09 kg/year) > Block D (2339.35 kg/year) > Block B (1932.53 kg/year) > Block C (685.85 kg/year) > Block J (315.83 kg/year).

**Figure 9:**
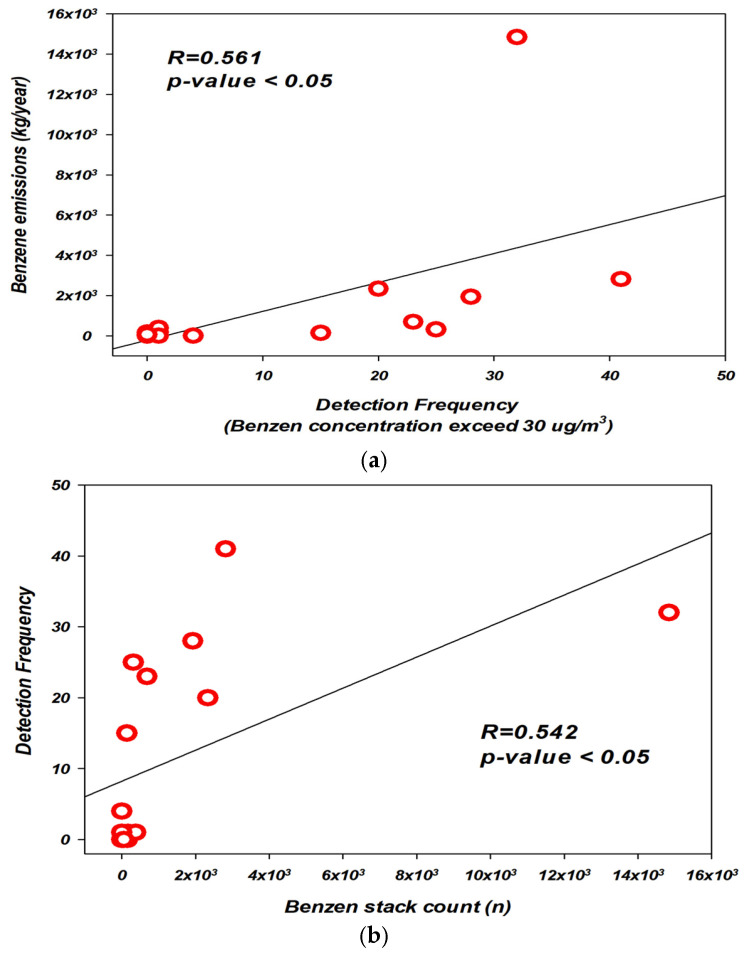

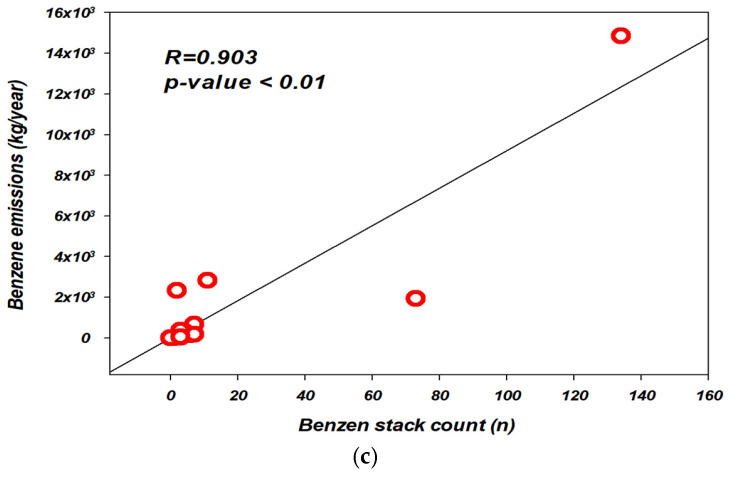
Comparison of benzene exceedance frequency with SEMS-based indicators in the Ulsan Petrochemical Complex. (**a**) Exceedance frequency vs. estimated benzene emissions (kg/year). (**b**) Exceedance frequency vs. number of benzene-handling stack count (n). (**c**) Benzene emissions (kg/year) vs. number of benzene- handling stack count(n).

Figure 9 illustrates the results of a correlation analysis between the frequency of high-concentration benzene exceedances, total benzene emissions, and the number of stacks handling benzene. The results showed a weak but statistically significant correlation between exceedance frequency and total emissions (R = 0.561, *p* < 0.05), as well as between exceedance frequency and the number of benzene-handling stacks (R = 0.542, *p* < 0.05).

These findings suggest that trace emissions may occur directly or indirectly from various production, storage, transfer, and treatment processes. Further process-level investigation is needed to precisely identify the sources. A study on the safety management status of the Yeosu Petrochemical Complex reported that many of the facilities are over 20–30 years old, increasing the potential for gas leakage [47]. Investigations into VOC exposure pathways have shown that most emissions occur at pipeline joints and connectors, often involving alkane compounds [48,49].

Although benzene is generally handled within sealed systems, it can be released during material transfers, equipment leaks, inventory evaporation, maintenance operations, or open handling in smaller facilities [50]. Kim et al. (2008) also reported that benzene levels in workers’ biological samples increased significantly during regular maintenance periods compared to routine operations [51].

According to the Chemical Safety Agency, 68.5% of total chemical releases in Korea occur within industrial complexes, and approximately 61% of these are fugitive emissions from facilities or processes that bypass emission control systems, particularly in non-stack sources, as identified by the Pollutant Release and Transfer Register (PRTR) [52].

## 4. Conclusions

Petrochemical complexes are large-scale industrial areas with numerous and diverse emission sources, making it challenging to identify specific pollutant origins using traditional two-dimensional monitoring methods. In this study, a three-dimensional approach employing Mobile-extraction Differential Optical Absorption Spectroscopy (Me-DOAS) was applied to assess benzene emissions across temporal, spatial, and situational dimensions within the Ulsan Petrochemical Industrial Complex in South Korea.

### 4.1. Monthly Analysis

The highest monthly average benzene concentrations (arithmetic mean) were observed in February, followed by January, April, and November (second campaign). The February mean was approximately 2.7 to 11.6 times higher than other months based on the arithmetic mean, and January showed a geometric mean 1.4 to 2.7 times higher than other months (*p* < 0.01). Although a direct comparison to the national annual standard (5 µg/m^3^) is limited, all twelve months exceeded this threshold based on arithmetic means, while four months (January, February, April, and November [2nd]) exceeded it based on geometric means.

### 4.2. Time-of-Day Variation

The highest hourly benzene concentrations were recorded during the fourth session in February (01:00–03:00), followed by the third session in February, the third session in April, and the fourth session in July. Geometric means were highest in the February fourth session, followed by April third, November (second) third, and February third sessions. The February third session showed 1.6 to 80.8 times higher arithmetic means and 1.3 to 17.2 times higher geometric means compared to other time periods (*p* < 0.01). Overall, the majority of high benzene concentrations occurred during the third and fourth sessions (night and early morning).

### 4.3. High-Concentration Hot Spots

A total of 179 exceedance events (based on a 30 µg/m^3^ threshold) were recorded. Block G exhibited the highest frequency (41 cases, ~23%), followed by F (32), B (28), J (25), C (23), D (25), and K (14). In Block G, the exceedances were most frequent during the fourth session (20 cases), followed by the third (11 cases), and five cases each during the first and second sessions. Seasonal analysis showed that exceedance events were most frequent in February (33 cases), November (second; 30 cases), January (28 cases), and December (16 cases), highlighting elevated benzene risks during the cold season.

### 4.4. Emission Source Characteristics

According to the 2022 Stack Emission Management System (SEMS) data, benzene emissions were higher in blocks with multiple processes and the presence of storage and production facilities. A weak but statistically significant correlation was found between exceedance frequency and both emission volume (R = 0.561, *p* < 0.05) and number of benzene-handling stacks (R = 0.542, *p* < 0.05).

This study demonstrated that benzene concentrations tend to be elevated during winter months and at night (22:00–03:00), and that hotspots are closely associated with areas where benzene-handling operations such as storage and manufacturing are present. By using Me-DOAS, this study offers a novel spatiotemporal framework for evaluating benzene emissions in industrial settings.

The findings provide a valuable foundation for designing more effective benzene management strategies tailored to time, location, and emission characteristics. While Me-DOAS, as an optical remote-sensing instrument, may exhibit limitations in precision compared to conventional canister sampling followed by GC-FID analysis, it is highly effective for real-time monitoring of spatiotemporal trends. Future efforts will focus on improving accuracy and precision through rigorous QA/QC protocols and comparative studies with established analytical methods.

### 4.5. Data Quality and Limitations

Benzene concentrations were measured using a mobile DOAS system equipped with an internal light source. Because of the intrinsically weak absorption features of benzene and the presence of potential spectral interferences, the retrieved concentrations should be regarded as semi-quantitative. Despite these limitations, the measurements revealed pronounced spatiotemporal patterns, with elevated concentrations observed during specific months, time intervals, and in downwind receptor areas. To ensure the robustness of the results, a rigorous multi-tiered QA/QC protocol was applied, including the removal of low-SNR outliers, false positives, and irregular fluctuations. The final dataset provided sufficient spatial and temporal resolution to delineate potential high-exposure zones and to support meaningful relative comparisons. Future research will aim to refine this framework by integrating advanced source apportionment methodologies and undertaking comprehensive modeling validation to improve the accuracy and interpretability of emission assessments.

## Figures and Tables

**Table 1:** Ambient air quality standards for benzene in selected countries.

Category Range	Country	Averaging Interval	Standard (µg/m^3^)
<5 µg/m^3^	France	Annual	2
	Israel	Annual	1.3
	Iraq	Annual	3
	Japan	Annual	3
	Israel	24 h	3.9
	New Zealand	Annual	3.6
	Scotland	Annual	3.25
	North Ireland	Annual	3.25
	Peru	Annual	4
	Sweden	Annual	Upper threshold 3.5
	Malta	Annual	Upper threshold 3.5
5 µg/m^3^	India, Lebanon, Russia, South Korea, Botswana, European Union, Colombia	Annual	5
	Albania	8 h	5
≤10 µg/m^3^	Russia	24 h	100
		20 min	300
	Syria	Annual	20
	Vietnam	1 h	22
		Annual	10
	Morocco	Annual	10
	South Africa	Annual	10
	Belarus	24 h	40
		Annual	10
	Cuba	20 min	1000

**Table 2:** Monthly benzene concentration in 2024.

Month	Mean	SD	Min	Max	GM	5th	25th	50th	75th	95th	*p*-Value	Post Hoc
Jan. ^a^	23.55	80.33	0.01	1132.51	8.09	1.06	4.59	7.89	13.64	77.66	0.01	b, c, d, e, f, g, h, i, j, k, l
Feb. ^b^	64.28	194.69	0.01	1955.38	5.13	0.26	1.41	3.62	11.96	451.14		a, c, e, f, g, h, i, j, k, l
Mar. ^c^	5.90	7.30	0.01	171.25	3.73	0.46	2.25	4.83	7.77	12.71		a, b, d, e, f, i, j, k, l
Apr. ^d^	20.35	50.31	0.01	588.31	5.44	0.48	2.40	5.16	10.10	116.74		a, c, e, f, g, h, i, j, l
May. ^e^	7.30	10.12	0.01	178.09	4.03	0.41	2.17	4.88	8.76	21.67		a, b, c, d, f, h, i, j, k
Jun. ^f^	5.51	7.65	0.01	97.78	3.02	0.32	1.58	3.58	7.22	14.09		a, b, c, d, e, g, h, i, j, l
Jul. ^g^	7.50	24.41	0.01	552.5	3.94	0.53	2.58	4.88	7.18	13.54		a, b, d, f, h, i, j, k
Aug. ^h^	6.26	12.05	0.01	214.60	3.53	0.50	2.18	4.08	6.40	16.64		a, b, d, e, f, g, j, k, l
Oct. ^i^	6.37	9.93	0.01	150.40	3.32	0.35	1.80	3.87	6.94	21.66		a, b, c, d, e, f, g, j, k, l
Nov. ^j^	6.45	5.59	0.01	104.11	4.54	0.71	2.94	5.53	8.72	13.90		a, b, c, d, e, f, g, h, i, k, l
Nov (2). ^k^	12.97	31.44	0.01	619.48	5.66	0.68	2.99	5.81	11.07	48.12		a, b, c, e, f, g, h, i, j, l
Dec. ^l^	10.37	25.74	0.01	361.01	3.98	0.39	1.92	4.42	8.61	36.81		a, b, d, f, h, i, j, k

Note: No measurements were conducted in September due to a technical malfunction of the Me-DOAS system; Jan.–Dec." is an abbreviation summarizing the months from January to December; The superscripts (from a to l) represent the months, and "post Hoc" refers to the post hoc test conducted using ANOVA analysis; SD refers to standard deviation, Min to minimum value, Max to maximum value, and GM to geometric mean.

**Table 3:** Characteristics of benzene-handling activities by block in the Ulsan Petrochemical Complex.

Block	Corporation	Benzene Used	Operation Process	Emission (kg/Year)	Total Stack Count (n)	Benzene Stack Count (n)	Storage Tank	Detection Frequency
A	#1	used	RTO (Regenerative Thermal Oxidizer)	22.38	9	3	-	N.D.
B	#2	used	Manufacture, Storage, RTO (Regenerative Thermal Oxidizer)	1932.53	109	73	Install	28
C	#3	used	Manufacture RTO (Regenerative Thermal Oxidizer)	685.85	12	7	-	23
D	#4	used	Manufacture VCU (Vapor Combustion Unit)	2339.35	22	2	-	20
E	#5	used	Supply of raw material Transfer of raw material A/C TOWER (Activated Carbon Tower)	0.066	6	1	-	N.D.
F	#6	used	Manufacture RTO (Regenerative Thermal Oxidizer) Vent gas treatment	14,844.33	149	134	-	32
G	#7, #8	used	REACTOR Fracking process	2819.09	15	11	-	41
H	#9	used	RTO (Regenerative Thermal Oxidizer) Manufacture	155.206	12	6		N.D.
I	#10	not used	-	0	-	-	-	1
J	#11	used	Storage of raw material	315.83	7	4	Install	25
K	#12	used	Manufacture CTO (Catalytic Thermal Oxidizer)	145.931	18	5	-	15
L	#13	used	TO (Thermal Oxidizer) RTO (Regenerative Thermal Oxidizer)	0	2	0	-	N.D.
M	#14	used	Manufacture RTO (Regenerative Thermal Oxidizer)	172.64	15	7	-	1
N	#15, 16	not used	-	0	-	-	-	4
O	#17	used	Manufacture, Storage VCU (Vapor Combustion Unit) RTO (Regenerative Thermal Oxidizer)	381	16	3	Install	1
P	#18	not used	-	0	-	-	-	1
Q	#19	used	Storage, Supply of raw material Melting, Fusion	60.976	12	3	Install	N.D.

Note: N.D. indicates “Not Detected”, signifying that the detection frequency was zero during the measurement period.

## Data Availability

The datasets used or analyzed during the current study are available from the corresponding author upon reasonable request.
